# Effects of Maternal Resveratrol Intake on the Metabolic Health of the Offspring

**DOI:** 10.3390/ijms22094792

**Published:** 2021-04-30

**Authors:** Purificación Ros, Jesús Argente, Julie A. Chowen

**Affiliations:** 1 Department of Pediatrics, Hospital Universitario Puerta de Hierro-Majadahonda, 28222 Madrid, Spain; prosmon@hotmail.com; 2 Department of Pediatrics, Universidad Autónoma of Madrid, 28029 Madrid, Spain; jesus.argente@uam.es; 3 Department of Endocrinology, Hospital Infantil Universitario Niño Jesús, Instituto de Investigación La Princesa, 28009 Madrid, Spain; 4 Centro de Investigación Biomédica en Red de la Fisiopatología de la Obesidad y Nutrición (CIBEROBN), Instituto de Salud Carlos III, 28029 Madrid, Spain; 5 Instituto Madrileño de Estudios Avanzados Food Institute (IMDEA), Campus of International Excellence, Universidad Autónoma of Madrid and Consejo Superior de Investigaciones Científicas (CSIC), 28049 Madrid, Spain

**Keywords:** maternal resveratrol, metabolic health, offspring, programming, sex differences

## Abstract

Maternal nutritional imbalances, in addition to maternal overweight and obesity, can result in long-term effects on the metabolic health of the offspring, increasing the risk of common non-communicable disorders such as obesity, diabetes and cardiovascular disease. This increased disease risk may also be transmitted across generations. Unfortunately, lifestyle interventions have shown reduced compliancy and limited efficacy. Resveratrol is a natural polyphenolic compound reported to have pleiotropic beneficial actions including a possible protective effect against the metabolic programming induced by poor dietary habits during development. However, studies to date are inconclusive regarding the potential metabolic benefits of maternal resveratrol supplementation during pregnancy and lactation on the offspring. Moreover, the responses to metabolic challenges are suggested to be different in males and females, suggesting that the effectiveness of treatment strategies may also differ, but many studies have been performed only in males. Here we review the current evidence, both in humans and animal models, regarding the possible beneficial effects of maternal resveratrol intake on the metabolic health of the offspring and highlight the different effects of resveratrol depending on the maternal diet, as well as the differential responses of males and females.

## 1. Introduction

**Figure 1:**
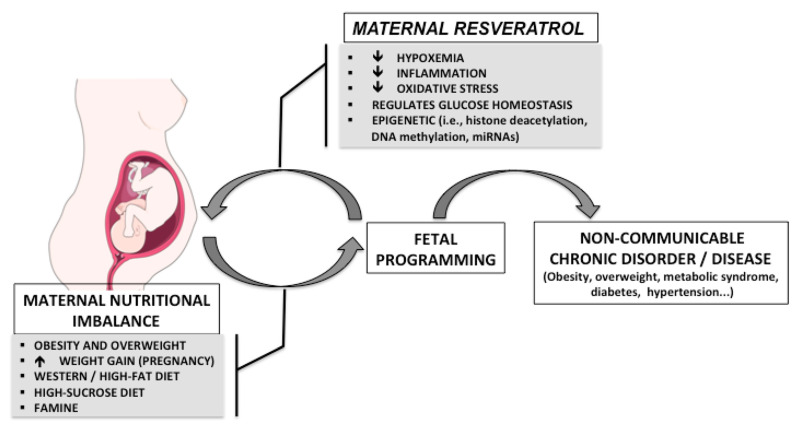
Possible programming effects of maternal nutritional imbalance during fetal and perinatal life and the potential beneficial effects of resveratrol.

The developmental origins of health and disease theory emphasizes the link between fetal and neonatal exposure to environmental factors and the development of metabolic and other disorders later in life [1,2,3,4,5,6,7,8,9]. This theory indicates that maternal nutrition during sensitive time periods of early development, prior to and during pregnancy and lactation, can strongly impact the offspring’s health later in life. Moreover, increasing evidence has shown that maternal nutritional imbalances, in addition to maternal overweight and obesity, may lead to long-term effects on the metabolic health of the offspring, increasing the risk of common non-communicable disorders and diseases such as obesity, diabetes and cardiovascular disease [10,11,12,13] (Figure 1) and that these effects can even be transmitted to future generations. Likewise, maternal resveratrol intake has also been shown to have effects on both the mother and fetus, with a possible interaction between maternal nutrition and resveratrol intake on the offspring’s long-term health, as depicted in Figure 1. Obesity is a prevalent metabolic disorder worldwide and the fact that the maternal obesogenic environment can lead to cyclical transgenerational transmission could contribute substantially to augmenting the obesity epidemic. Indeed, in 2016 the World Health Organization reported that in Europe 50% of women of childbearing age and 20%–25% of pregnant women were either overweight or obese [14]. Curtailing excessive weight gain during pregnancy has been shown to have health benefits for both the mothers and offspring, emphasizing that it is crucial to target all stages of development in order to reverse the obesity trend [15]. Unfortunately, lifestyle interventions, including diet and exercise, have shown low compliancy and limited efficacy.

**Figure 2:**
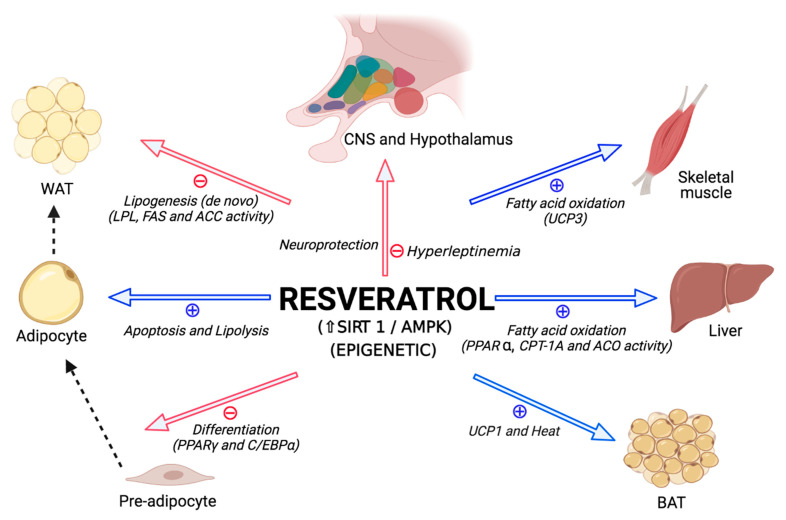
Proposed metabolic effects of resveratrol. ACC: acetyl-CoA carboxylase; ACO: acyl-CoA oxidase; AMPK: AMP-activated protein kinase; BAT: brown adipose tissue; C/EBP: CCAAT/enhancer-binding protein; CNS: central nervous system; CPT: carnitine palmitoyltransferase; FAS: fatty acid synthase; LPL: lipoprotein lipase; PPAR: peroxisome proliferator-activated receptor; SIRT: sirtuin; UCP 1 and 3: uncoupling protein 1 and 3, respectively; WAT: white adipose tissue. The red arrows represent inhibition and the blue arrows stimulation. The dashed black line indicates the direction of tissue changes. Figure modified from Aguirre L. et al. [20]. Created in BioRender.com.

An important area of investigation in the field of nutrition and metabolism is the search for dietary components and supplements that improve metabolic health, and one compound that has received increasing attention in recent years is resveratrol (3,4′,5-trihydroxystilbene). Resveratrol is a natural polyphenolic compound produced in a variety of plant species (i.e., grapes, peanuts, cocoa, berries) and has been shown to have anti-inflammatory [16], antioxidant [17], anti-obesogenic [18,19,20], anti-atherosclerotic [21] and antidiabetic effects [22,23,24] (Figure 2). Furthermore, this polyphenol is hypothesized to be potentially useful in protecting against factors during development that program susceptibility to metabolic syndrome [23,25,26] (Figure 2). However, different experimental models, as well as a wide range of doses, routes of administration and therapeutic periods, have led to inconclusive results regarding the potential metabolic benefits of maternal resveratrol supplementation during pregnancy and lactation [27]. Moreover, the responses to various metabolic challenges have been shown to be different in males and females [28,29,30,31,32] and this might suggest that the effectiveness of treatment strategies may also differ. Not only is there a sex difference in weight gain and adipose tissue accumulation and distribution in response to increased energy intake, but the secondary complications due to being overweight or obese also differ between males and females [33]. Unfortunately, little is known regarding whether long-term metabolism is affected differently in males and females in response to specific nutritional changes, including resveratrol intake, during early life.

Here we review the current evidence, both in humans and animal models, regarding the possible beneficial effects of maternal resveratrol intake on the metabolic health of the offspring. We have highlighted the differential effects of resveratrol depending on the maternal diet, as well as the differences between males and females in response to both dietary challenges and resveratrol.

## 2. Metabolic Effects of Maternal Nutritional Imbalances

In support of the developmental origins of health and disease theory, animal models and human studies have demonstrated that early environmental influences, including nutritional factors, can affect adult metabolic homeostasis [34,35,36,37]. Indeed, unbalanced nutrition during pregnancy and lactation has been shown to induce both short- and long-term effects on the metabolic health of the child, including an increased risk of obesity, diabetes and cardiovascular disease [36,38]. This indicates that modification of the nutritional supply to the fetus and newborn offers a window of opportunity to improve metabolic homeostasis of the offspring in later life, with effects on the hypothalamic–adipose axis and leptin signaling hypothesized to play a central role in this process [4,39].

### 2.1. Experimental Animal Studies

In experimental animal studies, maternal overnutrition and obesity increase the incidence of obesity in the offspring, with unfavorable programming of the appetite-regulating system in the hypothalamus shown to be involved [28,40]. Consistent with this, central leptin resistance has been observed in offspring from overfed or obese rodent mothers, as demonstrated by higher hypothalamic suppressor of cytokine signaling 3 (SOCS3) activation and blunted downstream signaling of leptin [40,41]. It is important to note that in rodents the initial postnatal weeks are critical for the formation of hypothalamic neurocircuits, with approximately postnatal day (PND) 20 being the end of the sensitive period for hypothalamic differentiation and development [41]. Tsai et al. showed leptin dysregulation, as indicated by an increase in plasma leptin/soluble leptin receptor (sOB-R) ratio, and increased retroperitoneal adiposity in adult male offspring from high-fat fed mothers [13].

Other maternal nutritional imbalances, such as a high-fat/high-sucrose diet during gestation, have been shown to affect the hepatic lipid profile in the offspring at 4 weeks of age, with some sex-specific effects [42]. Moreover, in rodents high-fructose consumption by dams during pregnancy and lactation are reported to led to sex-specific developmental programming of the metabolic syndrome phenotype in adult offspring, with this being more pronounced in males than in females [43]. Maternal high-fructose diet during gestation and lactation also impairs learning and memory performance in a sex-specific manner, due at least in part to an epigenetic mechanism of increased histone deacetylase 4 (HDAC 4) activity and suppression of neurogenesis in the hippocampus, with adult female offspring being more affected [12]. A maternal high-fructose diet is also reported to initiate neuro-inflammation in the hippocampus of adult female offspring [44]. A recent review analyzes the negative programming effects of maternal obesity, both in animal models and humans, emphasizing the five major mechanisms implicated to date: the gut–brain axis, inflammation, mitochondrial dysfunction, brain-derived neurotrophic factors and epigenomic effects [26]. 

Mal-programming of metabolism also occurs with maternal subnutrition. For example, in rodents maternal food restriction (50% caloric restriction during gestational days 12.5 to 18.5) was found to significantly decrease glucose tolerance in adult offspring, as well as to increase lipogenic gene expression in adipose tissue and adipocyte size [45]. Furthermore, a maternal low-protein diet can lead to lower birth weight, impaired glucose tolerance, and decreased fasting serum insulin levels in offspring at weaning [46]. Thus, it is not only overnutrition, but also insufficient or poor nutrition that determine future metabolic health of the offspring.

### 2.2. Studies in Humans

Consistent with animal studies, numerous epidemiological studies in humans have shown that metabolic programming of energy balance can be attributed to events during the very early stages of development [47,48]. In contrast to rodents, the sensitive period for metabolic programming in humans occurs predominantly in utero, during the third trimester of gestation, as well as the early neonatal period [49]. In line with this, maternal undernutrition during pregnancy and lactation has been shown to significantly increase the risks of metabolic syndrome, non-alcoholic fatty liver disease, and visceral fat deposition and dysfunction in the adult offspring [47,48,50,51]. Famine is a natural model for investigating the effects of an early-life poor nutritional environment on energy homeostasis in later life. One well known example of this is the Dutch winter famine (1944–1945) where maternal undernutrition during pregnancy was associated with higher prevalence of diabetes and disorders of lipid profiles, glucose intolerance and insulin resistance in adult offspring aged 50–59, with the outcome depending on both the gestational period when exposed to famine and the offspring’s sex [35,50,51]. For example, Lumey et al., found an association between maternal undernutrition and increased total cholesterol and triglycerides, but only in females [51].

In humans, maternal obesity during pregnancy and lactation is one of the most typical examples of early-life overnutrition that has been linked to increased risks of metabolic disorders in the offspring [36,38,52,53,54]. A growing body of evidence indicates independent associations between maternal obesity, excessive weight gain, as well as diet during pregnancy and lactation, with childhood adiposity and cardiovascular risk indicators [36,38]. It is important to emphasize that not only obesity, but also weight gain is of importance as an increase in gestational weight gain has been shown to be an independent predictor for increased body mass index (BMI), adiposity and cardiovascular risks in the offspring [54]. Accordingly, a meta-analyses recently identified a dose-response association between maternal BMI and childhood obesity of the offspring, with maternal obesity or overweight increasing the odds of obesity in the child by 264% and 89%, respectively [55]. There is also a well-established relationship between micronutrient deficiencies and fetal development and childhood health with the early Nutrition Research Project emphasizing the importance of nutrition and lifestyle before and during pregnancy, as well as during lactation and infancy [36].

There is strong evidence, both in experimental animal models and human studies, supporting the detrimental effects of an unbalanced maternal diet on the offspring’s metabolic health. This emphasizes the fact that the periods of gestation and lactation offer important opportunities to improve metabolic health in the offspring and that strategies against obesity should not only take into consideration interventions in adulthood, but should begin very early in life, including during the fetal and neonatal periods [15]. However, much more information is needed regarding how early metabolic programming is influenced by macronutrients, as well as by specific micronutrients and bioactive compounds.

## 3. Effects of Resveratrol

The beneficial effects of specific dietary bioactive compounds such as resveratrol on energy metabolism during postnatal life have been reported by different authors [24,35,56,57]. For example, Franco et al. showed that resveratrol (30 mg/kg/d), administered from 150 PND to 180 PND, reduced the increase in body weight, hyperphagia, visceral obesity, hyperleptinemia, hyperglycemia, insulin resistance and hypoadiponectinemia induced by an experimental model of early weaning in rats [18]. These same authors also reported that in adult offspring of mothers fed a HFD, postnatal resveratrol treatment completely normalized the hyperleptinemia and increases p-STAT3 levels in the hypothalamus, [58] suggesting that this compound improves at least some of the adverse effects of maternal HFD on leptin signaling.

Given the protective effects of postnatal resveratrol intake and its capacity to cross the placenta, along with its lack of teratogenicity as reported in most studies, the effects of maternal resveratrol supplementation during gestation and/or lactation on the dams and their offspring’s health has become a point of interest [23,25,26,59]. Indeed, the unbalanced redox state induced by factors that result in poor metabolic outcome in the offspring indicates a potential mechanism by which perinatal resveratrol could be beneficial due to its antioxidant capacity. Although several bioactive redox modulators have been proposed for use during gestation and pregnancy, resveratrol has attracted the most interest. Studies have confirmed a link between molecular targets and signaling pathways of resveratrol [i.e., sirtuin 1 (SIRT1), 5′ adenosine monophosphate-activated protein kinase (AMPK), estrogen receptor α (ERα) and mammalian target of rapamycin (mTOR)] and the physiopathology of metabolic syndrome-related disorders [60] that could help to shed light on the mechanisms underlying metabolic syndrome, as well as those involved in establishing long-term metabolic health and potential targets for its improvement.

### 3.1. Animal Studies

Various studies in experimental animal models support the hypothesis that maternal resveratrol intake exerts beneficial effects on the offspring’s metabolic health [13,29,30,31,61,62,63,64,65,66,67,68] (Table 1). Different early life insults, such as maternal gestational diabetes (MGD), obesity and nutritional imbalances, have been used to study the potential protective effects of resveratrol. As summarized in Table 1, the most commonly used experimental models for these studies involve rodents [13,29,30,31,61,65,66,67,68,69]. However, in an attempt to approximate human physiology, some studies have also been performed in nonhuman primates [62,63,64].

There is evidence supporting the beneficial health properties of maternal resveratrol from studies performed in embryos and fetuses. In Sprague Dawley rats, maternal resveratrol intake (100 mg/BW/day) during gestation [(from embryonic day (ED) 3 to ED12] was shown to prevent embryonic oxidative stress and apoptosis associated with diabetic embryopathy [61]. These authors also found that resveratrol significantly suppressed the activation of caspases in 12-day embryos of diabetic dams. In a genetic MGD mouse model (C57BL/KsJ-Lep^db/+^), maternal resveratrol (10 mg/kg/day) was found to reduce gestational diabetes mellitus symptoms in pregnant female mice and improved offspring development, through reducing the enzymatic capacity for glucose production in the fetus, which is most likely related to AMPK activation [68]. Other authors have evaluated the anti-hyperglycemic and anti-teratogenic capacity of maternal resveratrol (100 mg/kg/d from gestational days 8 to 12, when neurulation occurs) in a streptozotocin-induced diabetic Wistar rat model. They found that, on the 19th gestational day, resveratrol ameliorated the oxidative stress produced in the fetus by maternal hyperglycemia, and thus showing embryo-protective properties [65]. These studies evaluated the short-term effects of maternal resveratrol on the fetus or embryo in diabetic rodent models, showing favorable results on metabolic homeostasis and redox state. However, the offspring’s longer-term outcome and whether there were differences between the sexes are not specified (Table 1).

The effects of maternal resveratrol on metabolic parameters at weaning have also been analyzed. In 2017 Zou et al. reported that resveratrol supplementation (0.2%) in HFD-fed dams (C57BL/6J mice) during pregnancy and lactation protected against the harmful effects of maternal HFD on white adipose browning and thermogenesis in male offspring at weaning, accompanied by persistent beneficial effects in the protection against HFD-induced obesity and metabolic disorders [66]. We used a Wistar rat model where dams received a low-fat diet (LFD; 10.2% Kcal from fat) or HFD (61.6% Kcal from fat) during gestation and lactation to determine the effect of maternal resveratrol intake (2–2.5 mg/kg/day in drinking water) during pregnancy and lactation on the metabolism of the offspring at weaning and observed that these effects depend on the type of diet ingested by the mother and the offspring’s sex [30]. Of note, we used a low dose of resveratrol that can be easily ingested in a normal diet and based on its described hormetic property [24,69]. We found that resveratrol decreased body weight (BW) and adipose tissue (visceral and subcutaneous) mass at weaning in offspring from HFD-fed dams but had no effect on these parameters in the offspring from LFD-fed dams. This finding suggests that the potential protective effects of resveratrol during gestation/lactation may be maternal diet-dependent. This observation could be due to the fact that, as stated above, the effect of resveratrol is related to its interaction with the cellular redox state. Moreover, we found that female offspring were more globally affected by maternal resveratrol intake than males [30].

Long-lasting effects of maternal resveratrol on the offspring have also been reported. In Sprague Dawley rats, Liu Ta-Yu and colleagues investigated the effects of resveratrol, administered in drinking water (50 mg/L), and maternal HFD intake on the progeny and the response to postnatal HFD intake. They reported that on PND140, maternal resveratrol intake reduced lipogenesis and adiposity, increased lipolysis and ameliorated leptin resistance in male progeny [69]. They also found that maternal resveratrol protected against the maternal high-fat/high-sucrose-induced decrease in SIRT1 mRNA levels in retroperitoneal adipose tissue and normalized the plasmatic leptin/sOB-R ratio of the offspring. We recently reported that some of the metabolic changes that we observed at weaning are maintained in the adult offspring, and that they remain sex and maternal diet-dependent. Maternal resveratrol intake reduced serum cholesterol levels in both male and female adult offspring from HFD mothers, but increased it in adult female offspring from mothers consuming a LFD. Resveratrol also increased visceral adipose tissue (VAT) in maternal LFD offspring of both sexes but decreased it in male offspring from mothers on a HFD. Interestingly, maternal resveratrol intake shifted the distribution of VAT adipocyte size to a significantly higher incidence of large adipocytes, regardless of sex or maternal diet [31], suggesting an effect on adipocyte development. Liu Ta-Yu reported a decrease in visceral adipocyte size in 4-month-old male progeny from dams fed a HFHSD plus resveratrol [69]. In Sprague Dawley rats, maternal resveratrol supplementation (50 mg/L in drinking water, from pregnancy to lactation) is also reported to reduce the maternal HFD-induced retroperitoneal adiposity and the increase in leptin/sOB-R ratio in adult male offspring, while no data in females were reported. However, they found no effect of resveratrol on SIRT1 expression in the retroperitoneal adipose tissue of these animals [13]. Long-lasting effects of maternal resveratrol intake during only lactation (20 mg/kg/day), have been recently reported [67]. Maternal resveratrol consumption during lactation in dams fed a normal control diet was found to decrease plasma cholesterol levels in the male offspring at PND 273, through suppression of hepatic cholesterol biosynthesis and promotion of hepatic cholesterol uptake. Furthermore, a decrease in hepatic 3-hydroxy-3-methylglutaryl-CoA reductase and an increase in hepatic LDL receptor levels were also observed in the maternal resveratrol male offspring [67].

Maternal protein restriction during pregnancy is a well-established early life undernutrition model that is known to increase both maternal and offspring oxidative stress, leading to metabolic dysfunction. Vega et al. demonstrated that maternal resveratrol intake (20 mg/kg/day throughout pregnancy) partially prevented low-protein diet-induced maternal, placental and offspring oxidative stress and metabolic dysfunction at PND 110. They also showed sex differences in the modifications of triglyceride levels, liver reactive oxygen species (ROS) and fat depots in the offspring [29]. Taken together, studies in rodents suggest metabolic benefits of maternal resveratrol consumption in the offspring (i.e, decreased cholesterol levels, lipogenesis, adiposity, leptin resistance). However, to date differences in experimental protocols have led to inconclusive results.

Studies in non-human primates have also shown placental and fetal benefits in response to resveratrol supplementation when the mother is on a Western diet (36% calories from fat) during pregnancy. In an experimental model employing Japanese macaques, Roberts and colleagues [62] reported that supplementation with resveratrol during pregnancy (final concentration of 0.37% in a Western diet, from 3 months before the breeding season until gestational day 130) increased uterine artery blood flow volume and decreased placental inflammation and fetal liver triglyceride deposition. However, fetal pancreatic mass was found to be enlarged by 42%, with a 12-fold increase in proliferation as demonstrated by Ki67 immunohistochemistry [62]. This same group showed that consumption of a Western-style diet during pregnancy impaired offspring islet vascularization in Japanese macaques. Furthermore, when dams were put on a normal healthy diet, islet vascularization was normalized to control offspring levels, whereas resveratrol supplementation caused a significant increase in capillary density above controls [63]. These authors stated that due to the observed alterations in fetal pancreatic development and, until the long-term consequences of increased vascularization can be determined, resveratrol supplementation during pregnancy is not advised [62,63]. Indeed, although resveratrol has shown to be well tolerated in experimental animals with no major adverse effects, several studies reported that resveratrol could exert toxic effects, especially at high doses (≥50 mg/kg) [70]. Using Japanese macaques, O’Tierney-Ginn and colleagues studied the in utero influence of resveratrol (0.37% incorporated in the food) on the effects of HFD (35% fat) consumption prior to and throughout pregnancy on fatty acid uptake in placental explants. At gestational day 130 (term = 173 days), resveratrol stimulated placental DHA uptake capacity, AMPK activation and mRNA levels of fatty acid transporter protein 4, FATP-4, scavenger receptor CD36 and fatty acid binding protein [64]. These findings suggest that resveratrol could protect placental fatty acid uptake capacity and AMPK activity, a nutrient-sensing target, from the negative effects of maternal HFD. In summary, studies analyzing maternal resveratrol in non-human primates have shown an improvement in artery blood flow volume, placental inflammation and fetal liver triglyceride deposition along with a potential protective role against HFD.

### 3.2. Human Studies

Although the evidence in humans is limited, specific effects of resveratrol have been reported in ex vivo human placenta and adipose tissue samples [71,72]. Lappas et al. investigated the effects of resveratrol at different concentrations (50, 100 and 200 μmL/L) on insulin resistance and placental inflammation associated with gestational diabetes. They reported that at a concentration of 200 μmL/L, resveratrol is able to decrease placental inflammation as the expression of the pro-inflammatory cytokines tumor necrosis factor (TNF), interleukin (IL)-6 and 8 were reduced [71]. This same group also found that resveratrol (200 μmL/L) significantly reduced the expression of IL-6 and IL-8 in ex vivo human omental adipose tissue and placenta [72].

To our knowledge, limited human studies have analyzed the effects of resveratrol intake during pregnancy and lactation but none of them analyzed the offspring’s health outcome [73,74]. In these two studies resveratrol was used as an adjuvant treatment in pregnant women. Malvasi et al. used resveratrol in addition to D-chiro-inositol and myo-inositol in overweight pregnant women with an elevated fasting glucose, finding an improvement in glucose levels, LDL cholesterol and triglycerides [73]. On the other hand, Ding and colleagues analyzed the effect of resveratrol as an adjuvant treatment of oral nifedipine to attenuate hypertensive symptoms [74].

## 4. Sex Differences in Response to Perinatal Changes

We know that males and females have metabolically different responses not only to early nutritional changes [75] but also to stressful conditions during early life [76,77] and that the sexually dimorphic responses to these early interventions often vary according to age [75,76]. However, much is yet to be learned about the long-term differences between males and females in response to early nutritional changes as many studies have only been performed in one sex, and this is normally males. To our knowledge, only three studies analyzing the effects of maternal resveratrol have focused on sex differences. Vega et al. showed clear sex differences in the response to maternal resveratrol intake as decreased triglycerides and liver ROS was observed in male but not in female offspring of low-protein diet-fed dams, with no effects in those on a control diet [29]. Ros et al. found sex differences in both the short- and long-term responses to an unbalanced maternal diet with or without resveratrol. At weaning, a decrease in BW, leptin and fat depots was observed in the offspring from HFD dams plus resveratrol when compared to those from HFD mothers, with females being more affected by resveratrol than males [30]. Maternal resveratrol intake was also found to decrease the visceral fat depot, but only in adult male offspring from HFD mothers [31]. 

## 5. Conclusions

Collectively, these studies indicate an effect of maternal resveratrol intake on the offspring. Although the mechanism of action of resveratrol is not completely understood, it has been hypothesized that it can induce epigenetic modifications. It is important to emphasize that the beneficial effects of resveratrol as a therapeutic intervention may be affected by many factors, such as the baseline health status of the subjects, nutritional habits, resveratrol dose and the intervention period. Although resveratrol is reported to be well tolerated and safe, caution must be exerted as abnormal pancreatic development and vascularization has also been reported in the offspring of non-human primate mothers consuming resveratrol. However, different study designs and objectives, as well the large variability in the dose, route and timetable of resveratrol administration, make it difficult to reach robust conclusions about the health benefits of resveratrol before considering it for human therapeutic use during pregnancy and/or lactation. In addition, the evidence concerning offspring outcome remains scarce even in animal models and some of these studies have been performed only in males. This is of great importance, as we know that the effects of early nutritional modifications can be sex-specific and data to date indicate that the response to early resveratrol also varies according to sex. Therefore, further and more homogeneous studies are needed to determine how resveratrol exposure during gestation and lactation affects long-term health with both the sex-specific responses and the type of diet ingested taken into consideration.

## Figures and Tables

**Table 1:** Experimental studies in pregnant animal models supplemented with resveratrol. AMPK: 5′ adenosine monophosphate-activated protein kinase. BAT: brown adipose tissue. BW: body weight. CD: control diet. DHA: docosahexanoic acid. ED: embryonic day. F: female. FAT: fat mass. GD: gestational day. Gest: gestation. HFD: high-fat diet. HFHS: high-fat high-sucrose diet. HMGCR: hydroxy-3-methylglutaryl-CoA reductase. Hth Hyperlep: hypothalamic hyperleptinemia. Lact: lactation. LFD: low-fat diet. LPD: low-protein diet. M: male. NS: not specified. PND: postnatal day. Preg: pregnancy. RPT: retroperitoneal tissue. ROS: reactive oxygen species. SCAT: subcutaneous adipose tissue. S-D: Sprague Dawley. SIRT1: Sirtuin 1; STZ: streptozotocin. TG: triglycerides. VAT: visceral adipose tissue. W: weeks. WAT: white adipose tissue. Wi: Wistar rat. WD: Western diet. * Genetic gestational diabetes model C_57_BL/KsJ-Lepdb/+ (db/+). ** C57BL/Gj mice. ↓ decreases, ↑ increases.

Model	Species/Sex	Resveratrol Dose and Route	Time	Age at Outcome	Offspring Outcome	Reference
Maternal diabetes	Rat (S-D)/NS	100 mg/BW/d	3–12 d GA	ED 12	↓ oxidative stress and apoptosis	[61]
Maternal diabetes	GDM mouse */NS	10 mg/BW/d	4 w pre-preg. and gest.	PND 1	↓ BW and ↑ AMPK	[68]
Maternal diabetes	Rat (Wi)/NS	100 mg/BW/d	Gestation (8–12 d)	GD 19	↓ oxidative stress	[65]
Maternal LPD/CD	Rat (Wi)/M&F	20 mg/BW/d	Gestation	GD 19 and PND 110	Sex differences TG and ROS	[29]
Maternal HFD	Mouse **/M	200 mg/BW/d	Preg to PND21	PNDs 21 and 98	↑ BAT browning WAT	[66]
Maternal HFD/LFD	Rat (Wi)/ M and F	50 mg/dl in drinking water	Preg to PND21	PND 21	Sex differences ↓BW, Lept, VAT, SCAT	[30]
Maternal CD	Rat (Wi)/M	20 mg/BW/d	Lactation	PND 252	↓Plasma cholesterol ↓HMGCR	[67]
Maternal HFHS/CD	Rat (S-D)/M	50 mg/dl in drinking water	Preg to PND21	PND 180	↓BW, adiposity regulates SIRT 1 in RPT Lipid modulation	[70]
Maternal HFD	Rat (S-D)/M	50 mg/dl in drinking water	Preg to PND21	PND 120	↓ RP adiposity Improves leptin dysregulation	[13]
Maternal HFD/LFD	Rats (Wi)/ M and F	50 mg/dl in drinking water	Preg to PND21	PND 150	VAT ↓in HFD↑ in LFD ↑ VAT adipocyte size	[31]
Maternal WD	Japanese macaque/NS	+0.37% in WD	3m pre-G to 130 GA	ED130	↓ liver lipid and placental inflammation	[62]
Maternal WD	Japanese macaque/NS	0.37% in WD	3m pre-G to 130 GA	ED 130	↑↑ fetal islet vascularity	[63]
Maternal HFD/CD	Japanese macaque/NS	0.37% in WD	3m pre-G to 130 GA	ED 130	↑ Placental fatty acid uptake (DHA)	[64]
